# A homologous gene of Os*REL2/ASP1, ASP-LSL* regulates pleiotropic phenotype including long sterile lemma in rice

**DOI:** 10.1186/s12870-021-03163-7

**Published:** 2021-08-21

**Authors:** Tingkai Wu, Asif Ali, Jinhao Wang, Jiahe Song, Yongqiong Fang, Tingting Zhou, Yi Luo, Hongyu Zhang, Xiaoqiong Chen, Yongxiang Liao, Yutong Liu, Peizhou Xu, Xianjun Wu

**Affiliations:** State Key Laboratory of Crop Gene Exploration and Utilization in Southwest China, Rice Research Institute, Sichuan Agricultural University, 611130 Chengdu, China

**Keywords:** Rice, Aberrant spikelet, Long sterile lemma, Pleiotropic phenotype, Gene cloning, Relative expression

## Abstract

**Background:**

Panicle is a harvesting organ of rice, and its morphology and development are closely associated with grain yield. The current study was carried on a mutant screened through an EMS (ethyl-methane sulphonate) mutagenized population of a Japonica cultivar Kitaake (WT**)**.

**Results:**

A mutant, named as *asp-lsl* (*aberrant spikelet-long sterile lemma*), showed a significant decrease in plant height, number of tillers, thousand-grains weight, seed setting rate, spikelet length, kernel length and effective number of grains per panicle as compared to WT. *Asp-lsl* showed a pleiotropic phenotype coupled with the obvious presence of a long sterile lemma. Cross-sections of lemma showed an increase in the cell volume rather than the number of cells. Genetic segregation analysis revealed its phenotypic trait is controlled by a single recessive nuclear gene. Primary and fine mapping indicated that candidate gene controlling the phenotype of *asp-lsl* was located in an interval of 212 kb on the short arm of chromosome 8 between RM22445 and RM22453. Further sequencing and indels markers analysis revealed *LOC_Os08g06480* harbors a single base substitution (G→A), resulting in a change of 521st amino acid(Gly→Glu. The homology comparison and phylogenetic tree analysis revealed mutation was occurred in a highly conserved domain and had a high degree of similarity in Arabidopsis, corn, and sorghum. The CRISPR/Cas9 mutant line of *ASP-LSL* produced a similar phenotype as that of *asp-lsl*. Subcellular localization of ASP-LSL revealed that its protein is localized in the nucleus. Relative expression analysis revealed *ASP-LSL* was preferentially expressed in panicle, stem, and leaves. The endogenous contents of GA, CTK, and IAA were found significantly decreased in *asp-lsl* as compared to WT.

**Conclusions:**

Current study presents the novel phenotype of *asp-lsl* and also validate the previously reported function of *OsREL2 (ROMOSA ENHANCER LOCI2)*, / *ASP1(ABERRANT SPIKELET AND PANICLE 1).*

## Background

Rice is the world’s main food crop, and its yield is essential for food security. Spikelet development is one of the most important traits for yield. Rice is considered a model crop for monocotyledons due to its unique structural unit of spikelet compared to dicotyledons. Grasses spikelet consists of two sterile glumes (rudimentary glumes), empty glumes, and a pair of fertile glumes that are further called lemma and palea [1, 2]. According to previous studies, spikelet development can be roughly divided into the following eight stages: the first stage (Sp1) includes the formation of the incomplete rudimentary glume primordia. From second to fourth stage (Sp2-Sp4), glume primordium differentiates into lemma and palea primordium. The formation of lodicule and stamen occur at the fifth (Sp5) and sixth (Sp6) stage of spikelet development, respectively. The seventh (Sp7) and eighth (Sp8) stages involve the formation of carpel primordium and development of ovule and pollen, respectively [3]. So far, numerous genes have been cloned that function at different stages of spikelet development. Mutants with abnormal spikelet development showing hindrance at various stages can be used as important materials for studying molecular mechanism.

In terms of floral organs development, there are certain differences between monocotyledonous and dicotyledonous plants. However, homologous sequence alignment and transgene analysis showed that ABCDE model of dicotyledons applies to monocotyledons to a certain extent. Hence, above-mentioned model can also be applied to understand developmental mode of rice floral organs [4–11]. According to ABCDE model, so far, five types of MADS-box genes have been cloned and divided based on their function. These floral genes e.g., A, B, C, D and E regulate different independent or coordinated actions for a final phenotype of floret.

According to ABCDE model of rice, contrary to Arabidopsis, class A genes are mainly expressed in the outer two whorls of floral organs e.g., *OsMADS14* [12, 13], *OsMADS15* and *OsMADS18* [14] are all AP1 (ACITVATOR PROTEIN 1)-like genes [15, 16]. Exceptionally, *OsMADS14* expresses in spikelet meristem (SM), inner lemma, palea, pistil, and stamen at maturity [17]. *OsMADS14* affected the flowering period, and its overexpression leads to early flowering in rice [18]. *OsMADS15* normally expressed in lemma and lodicules, and mutants of *OsMADS15* exhibited elongation of inner and outer glumes [19]. *OsMADS18* has transcripts in all floral tissues and affected the flowering period. It’s overexpression caused early flowering but RNAi-silencing did not affect the phenotype compared with its wild type (WT) [20].

In rice, class B genes include e.g., *OsMADS2, OsMADS4*, and *OsMADS16/SUPERWOMAN1 (SPW1)* [21]. These are also conserved in angiosperms. *OsMADS2* and *OsMADS4* are homologous to the Arabidopsis PI *(PSEUDOGENE)* gene, and OsMADS16 is homologous gene of Arabidopsis AP3 gene [22, 23]. The transgenic plants of *OsMADS2* obtained by gene silencing showed abnormal elongation of lodicule [24]. *OsMADS2* played a key role in the development of rice panicle by controlling the function of lodicule and stamens [25]. Loss of function of *OsMADS4* triggered lodicules to exhibit a palea-like morphology, and stamens exhibited a carpel-like morphology. Similarly, loss of function of *OsMADS16* caused the stamens and lodicules to transform into carpels and palea-like structures, respectively [26].

In rice, class C genes include e.g. *OsMADS3* [5], *OsMADS58* [27] and *DROOPING LEAF (DL)* [28]. Among them, *OsMADS3* and *OsMADS58* are homologous genes of Arabidopsis AG gene[29], and DL is homologous to Arabidopsis *CRC (CRABSCLAW)*. *OsMADS3* normally expressed in carpels and stamens [30], which mainly controls the development of stamens [31] and inhibits lodicule development. Loss of function of *OsMADS3* showed decisive loss of floral meristem (FM), and stamens turn into a pulp-like structure. *OsMADS58* mainly controls the development of FM and its RNAi transgenic plant produced cup-shaped carpel [32]. *DL* regulates the development of FM, and a mutation in *dl* transformed the carpel into stamens [27].

In rice, class D genes mainly refer to *OsMADS13*, which is the STK (SERINE/THREONINE KINAS) gene of Arabidopsis and homologous to *FBP7 (F-BOX PROTEIN 67)/FBP11* of petunia. *OsMADS13* is only expressed in the carpel and mainly concentrated in the ovule. [30–32].

In rice, E genes include several types of genes e.g., *SEP (SEPALLATA), LOFSEP (LOSS OF TRANSCRIPTION FACTOR OF SEP)*, and *AGL6 (AGAMOUS-LIKE 6)* [33, 34]: *OsMADS7* (*OsMADS45*) and *OsMADS8 (OsMADS24)* are homologous to Arabidopsis *SEP* [35, 36]; while, LOFSEP include *OsMADS1/LEAFY HULL STERILE 1 (LHS1)* [37, 38]. *OsMADS5 (OsM5)* and *OsMADS34/PANlCLE PHYTOMER2 (PAP2*) are included in *AGL6* and mainly refers to *OsMADS6/MOSAIC FLRALORGANS 1 (MFO1)* [39]. In rice, the *LHS1* regulates the development of lemma and lemma coupled with the development of FM. Its ectopic expression can lead to the elongation of the spikelet [38, 40–42]. *OsMADS6* regulates the development of FM and floral organs and interacts with *DL* to jointly regulate the development of palea, making palea to a lemma-like structure [43]. *OsMADS34* has pleiotropic effect and its mutation not only turns the SM abnormal but also transformed the rudimentary glume into an elongated palea [37, 44].

Genes such as *OsMADS34* and *G1* can regulate the characteristics of rice glume development, and its mutations caused the glume to exhibit the phenotype of a lemma-like structures [45]. *MULTI-FLORET SPIKELET2 (MFS2)* encodes a MYB transcription factor and controls the identity of palea. *MFS2* is a transcriptional repressor (TRP) and interacts with TOPLESS (TPL)-related proteins and forms a complex; as a result number of floral organs were increased in *mfs2* [46]. Os*REL2 (ROMOSA ENHANCER LOCI2)*, orthologous to Arabidopsis TPL, showed a pleiotropic effect including long sterile lemma and defects in panicle heading [47]. Similarly, *OsLIS-L1 (LISSENCEPHALY TYPE-1-LIKE-1)* encodes a protein with WD (Trp-Asp) domain repeats and revealed semi-dwarfism and defects in fertility [48]. *ASP1 (ABERRANT SPIKELET AND PANICLE 1)* encodes a TPL protein and control pleiotropic phenotype, including change in the meristem, phyllotaxy, and branching meristem by the intervention of auxin [49].

In this study, we have screened a mutant created by EMS mutagenized population of a wild type cultivar Kitaake (WT). Phenotype, genetic, and expression analysis revealed this gene is homologous to already reported *REL2* and *ASP1.* Although similar phenotypes have already been reported in *asp1*, but presence of some different phenotypes especially long sterile lemma encouraged us to study further. Our findings not only validate pleiotropic effect of *ASP1* and *OsREL2* but also present some more phenotypic defects e.g., dwarfism, spikelet shortness and long sterile lemma, that we are reporting for first time.

## Results

### The phenotypic observation and measurement of agronomic traits revealed pleiotropic effect of *ASP-LSL*

**Fig. 1:**
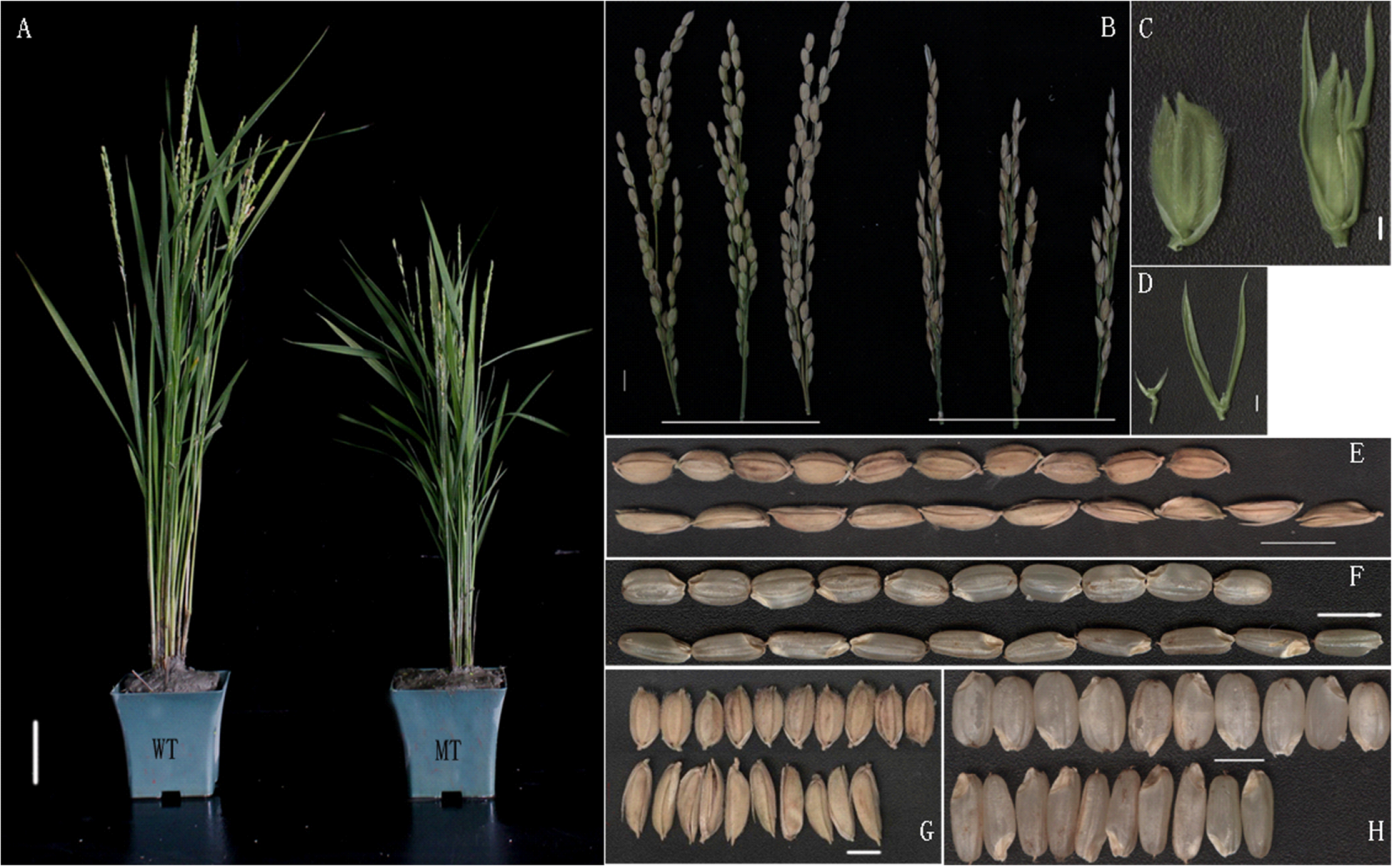
The phenotype of WT and *asp-lsl*. **A** The phenotype of WT (left) and *asp-lsl* (right). **B** The panicle of WT (up) and *asp-lsl* (up). **C** Spikelet of WT (left) and *asp-lsl* (right). **D** Sterile lemma of WT (left) and *asp-lsl* (right) **(E)** Horizontal view of mature grain of WT (up) and *asp-lsl* (down). **F** Horizontal view of kernel of WT (up) and *asp-lsl* (down). **G** Vertical view mature grain of WT (up) and *asp-lsl* (down). **H** Vertical view of kernel of WT (up) and *asp-lsl* (down). *asp-lsl* (down)

Data of agronomic traits of *asp-lsl* and WT (Kitaake) were measured and results revealed that plant height, length of panicle, grain length, number of seeds per panicle, thousand-grain weight, seed setting rate and length of sterile lemma were significantly different in *asp-lsl*. Compared with its WT, the *asp-lsl* has reduced plant height by 23.2 %, panicle length by 21.1 %, seed setting rate by 29.2 %, 1000-kernel weight by 28.9 %, spikelet length by 34.4 %, and kernel length by 22.3 %. The most prominent spikelet variation of *asp-lsl* mutant was its increased and widened sterile lemma, as compared to its WT (Fig. 1). The glume length and width were 4.7 and 2.7 times increased in *asp-lsl* than that of WT, respectively. Protective glumes accounted ~ 1.9 % of the thousand-grain weight in *asp-lsl*. After removing glumes, compared with its WT, *asp-lsl* caryopsis became slender and thousand-grain weight decreased by 33.1 % (as shown in Table 1). It indicates that changes in grain type of *asp-lsl* led to the decrease in thousand-grain weight, and ultimately decrease in yield.

**Table 1:** Measurement of agronomic traits of *asp-lsl *and WT

Material	PMD	PH (cm)**	PL (cm)*	SR (%) **	GL (mm)**	GW (mm)*	GL-WR*
KTK	89 ± 1	62.3 ± 1.52	13.3 ± 1.42	80.4 ± 0.04	7.1 ± 0.04	3.49 ± 0.03	2.04 ± 0.14
*asp-lsl*	96 ± 1	47.8 ± 1.11	10.5 ± 1.37	56.8 ± 0.05	8.77 ± 0.04	2.72 ± 0.02	3.22 ± 0.16

*PMD* Plant maturity days, *PH* Plant height, *PL* Plant length, *SR* seed setting rate, *GL* Grain length, *GW* Grain weight, *GL/GW* Grain length/Grain width.

*indicates the significance at 0.05 level and

**indicates the significance at 0.01 level

### *asp-lsl* showed delayed germination and decreased seed viability

**Fig. 2:**
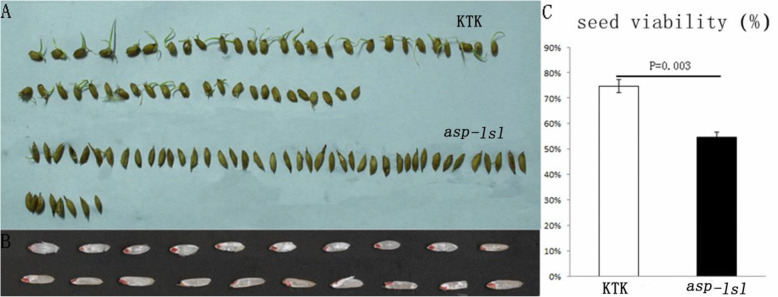
Germination and viability analysis of WT and *asp-lsl*. **A** The seeds germination of WT (up) and *asp-lsl* (down). **B** The grains of WT (up) and *asp-lsl* (up). **C** The seed viability of WT (left) and *asp-lsl* (right)

Compared with the WT seeds, the *asp-lsl* seeds were slender but not filled properly. Observation and statistics of germination record revealed not only the emergence rate of *asp-lsl* was lower but the germination was also significantly delayed. The germination rate of *asp-lsl* on the third day of germination was only 8 %, which was significantly lower than 72.3 % of its WT. The overall germination rate of *asp-lsl* was 69 % that was significantly lower than WT (88.3 %). Results of germination assay revealed that seed viability was significantly decreased in *asp-lsl* (Fig. 2).

### Analysis of inter-node length of stem

**Fig. 3:**
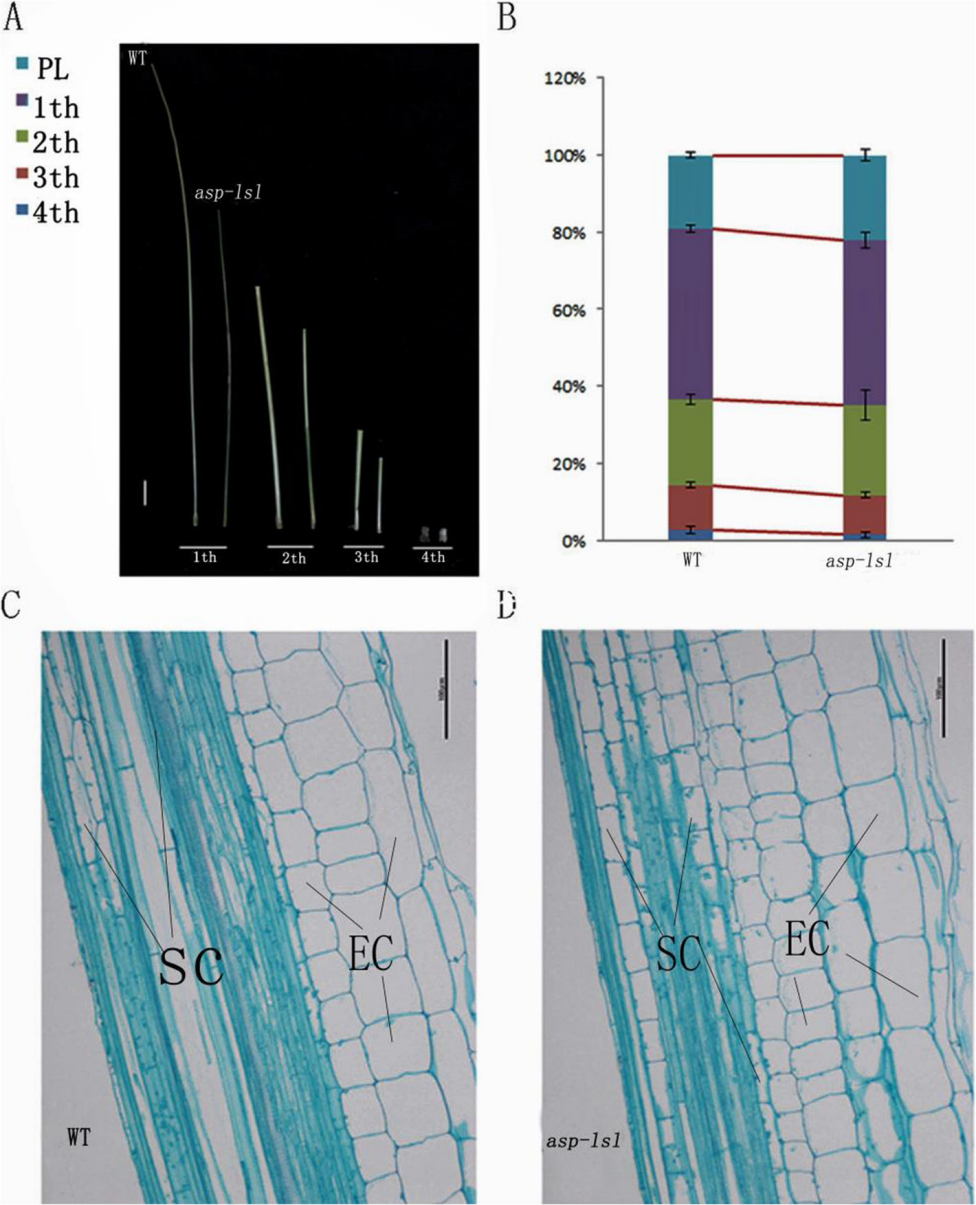
Observation and analysis of internodal distance of WT and *asp-lsl*. **A** The comparison of each respective internode of WT and *asp-lsl*, from left to right: first, second, third and fourth internode of WT and *asp-lsl*, respectively. bar=5cm** (B)**The column showing comparative length of four internodes at maturity. **C** WT transverse sections of the first internode, bar=50μm. **D** *asp-lsl*transverse sections of the first internode, where SC: silicide cell, EC: epidermis cell and bar=50μm

To further analyze of dwarfing of *asp-lsl*, we measured each stem internode at the maturity stage. Comparison of results showed the first, second, third, and fourth internodes of *asp-lsl* were shorter in length than those of WT (Fig. 3A). The relative change in length of the first, second, third, and fourth internode between *asp-lsl* is given in Fig. 3B. Among them, the difference in the first internode was more significant (Table 2). Analysis of the internode length of *asp-lsl* and WT revealed that dwarfing was mainly caused by the shortening of internodal distance.

**Table 2:** Analysis of internodal distance of *asp-lsl* and WT,

Internode length	WT (cm)	Proportion(%)	MT(cm)	Proportion(%)
1st	31.7 ± 0.6	54.9	23.4 ± 3.3**	53.5
2nd	15.7 ± 0.86	27.2	12.77 ± 2.10	30.9
3rd	8.4 ± 0.44	14.6	5.63 ± 2.61	13.6
4th	1.9 ± 0.57	3.3	0.8 ± 0.23	2.0

**indicates the significance at 0.01 level

### Spikelet morphology and fertility observation of *asp-lsl*

**Fig. 4:**
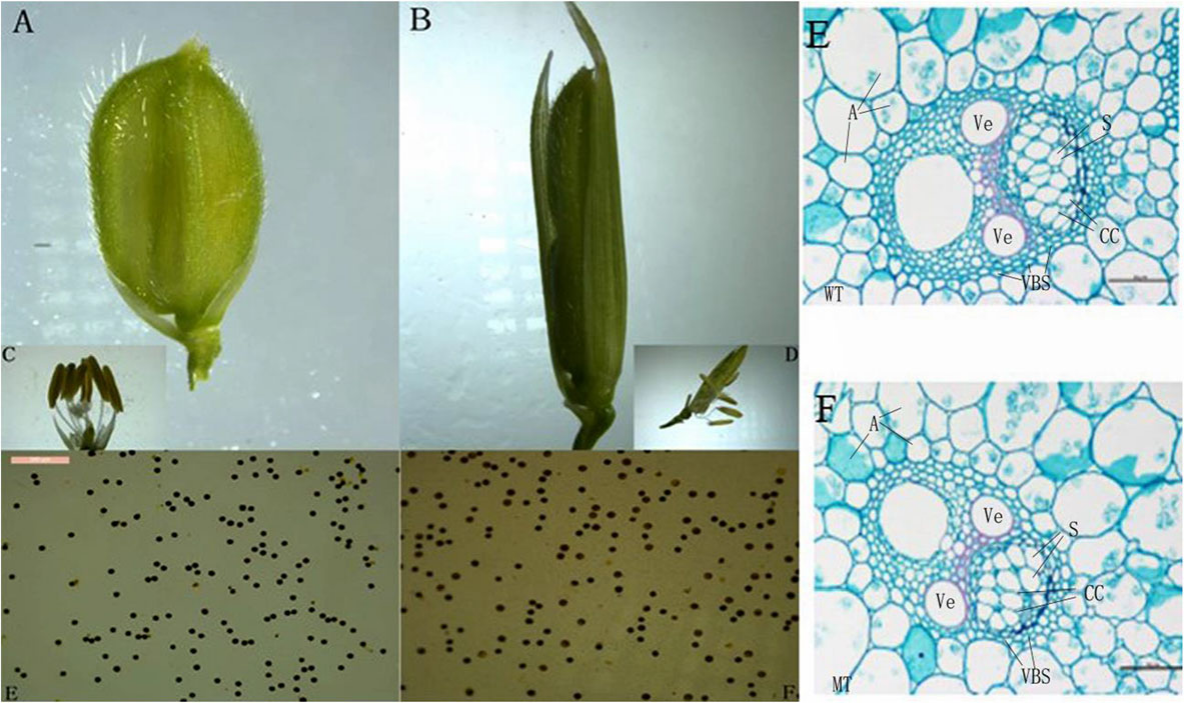
The sterile lemma morphology and Potassium Iodide staining of WT and *asp-lsl* (**A**) The sterile lemma of WT. **B** The sterile lemma of *asp-lsl* (**C**) Pollen grains of WT (**D**) Pollen grains of *asp-lsl* **E)** The paraffin sections of WT **(F)** and *asp-lsl.* Where A: amyloplast, Ve: vessel, VBS: vascular bundle sheath and CC: companion cells

The spikelet of *asp-lsl* has a similar floret structure to WT except a longer sterile lemma (Fig. 4A and B). Pollen viability was checked using KI-I_2_ staining, and results revealed no significant changes in fertility between WT and *asp-lsl* (Fig. 4C and D). To further look down minor changes in lemma morphology, paraffin cross-sections were prepared (Fig. 4E and F). The results of lemma morphology observation revealed the presence of larger cell volume. However, there was no significant change in the number of cells of sterile lemma. So, larger lemma was produced as result of the change in cells volume rather than the number of cells.

### *asp-lsl* showed significant changes GA, CTK, and IAA phytohormones

**Fig. 5:**
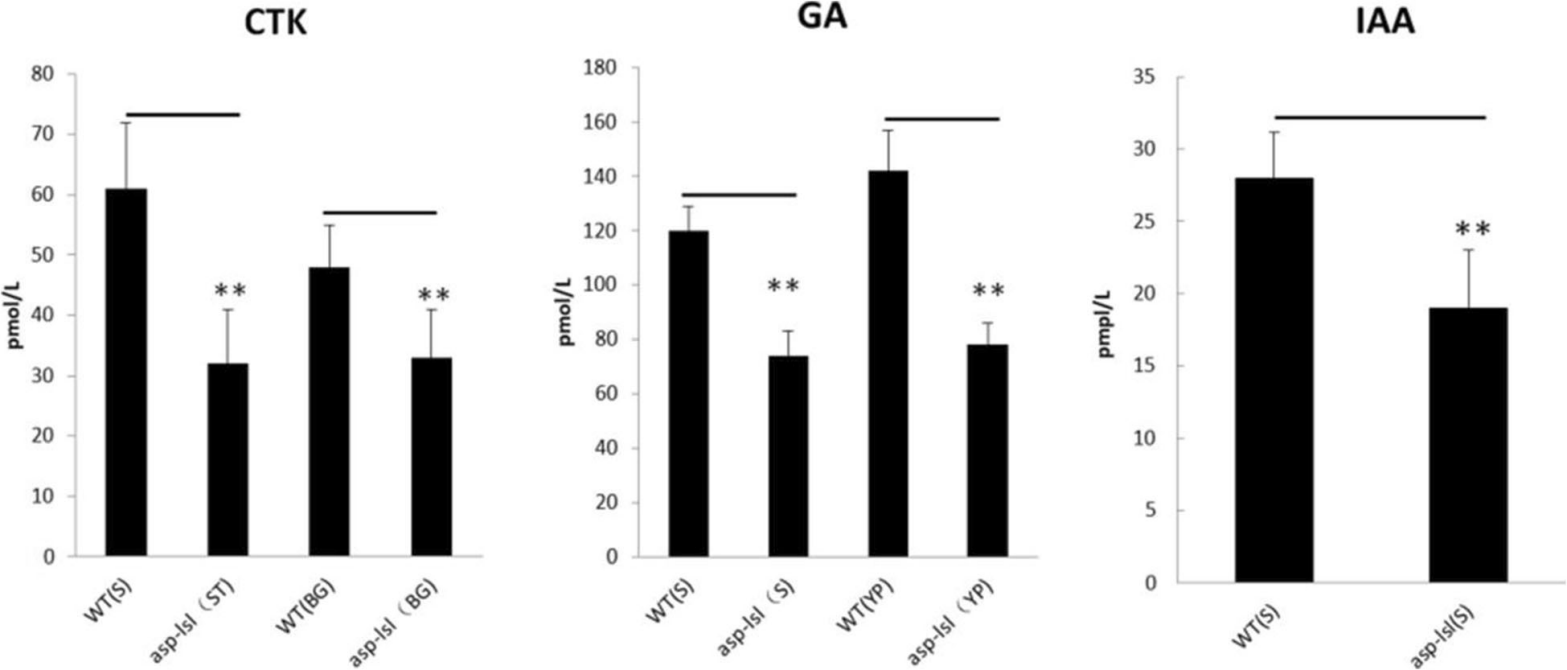
Endogenous hormone contents of WT and *asp-lsl.* S: stem, YP: young panicle, BG: before grouting, MS: mature stage

In order to compare changes in the shape of *asp-lsl* architecture, we determined the relevant hormones e.g., GA (Gibberellins), IAA (Indole acetic acid), and CTK (cytokinin) content in stem and panicle. The results showed GA, CTK, and IAA of *asp-lsl* had significant changes as compared with its WT (Fig. 5). GA, CTK, and IAA of *asp-lsl* were found significantly lower in stem and young panicle than that of WT.

### A single recessive gene controls the phenotype in *asp-lsl*

Using Yixiang 1B and KTK as male parents and *asp-lsl* as female parent, were crossed to get two mapping populations (*asp-lsl* *YXB and *asp-lsl* *KTK). Both populations were observed for phenotypes in F_1_ and F_2_. The results showed that the phenotype of all F_1_ plants were consistent with the WT phenotype. F_1_ were selfed to get F_2_ progeny; in later one, plants appeared with the both (WT and *asp-lsl*) type of phenotype. χ2 (Chi-square) test of segregation ratio between of WT vs. *asp-lsl* was in accordance with the Mendelian ratio of 3:1 (Table 3). The phenotypic segregation ratio of both F_2_ population can validate that mutant phenotype is controlled by a single recessive gene.

**Table 3:** The genetic segregation ratio of *ASP-LSL*

Cross	F_1_	F_2_			χ2(3:1)
		Total No. of plants	No. of mutant type plants	No. of wild type plants	
*asp-lsl* /KTK	Normal	570	128	442	1.17^a^
*asp-lsl* /YXB	Normal	670	174	496	0.67^a^

^a^indicates the significance at 0.05 level

### Primary and fine mapping of *ASP-LSL*

**Fig. 6:**
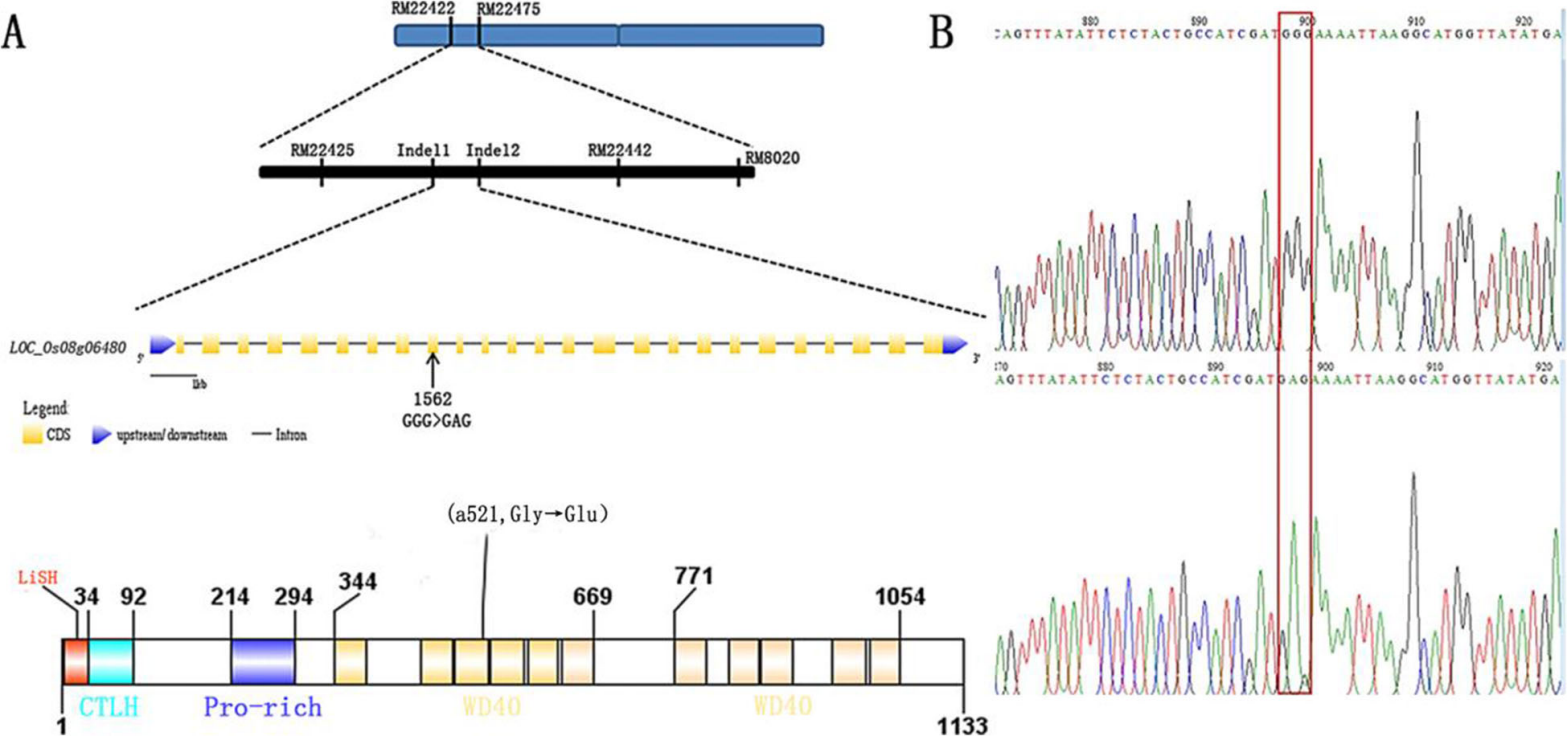
Gene mapping of *ASP-LSL* (**A**) Primary gene mapping of locus on short arm chromosome 8, Yellow line showed the structure of candidate gene, the Protein structure of candidate gene. **B** The chromatogram of WT and *asp-lsl* showing SNP

An F_2_ segregated population, constructed from the cross of *asp-lsl* and Yixiang 1B was used for gene mapping. Primarily, 456 pairs of SSR markers were used for primary gene mapping and locus was initially positioned between RM22418 and RM22529 (Fig. 6). In order to further locate, different Indel markers were applied and the interval was narrowed down to a region of 212 kb between Indel-1 and Indel-2. Twenty-six candidate genes were present in an interval of 212 kb. Analyzing all genes in this interval, it was found that the *LOC_Os08g06480* contains an SNP. Using WT and *ASP-LSL* cDNA as templates, the gene was amplified and sequenced. An SNP was detected that changed cDNA (1562nd nucleotide) of *LOC_Os08g06480* from G (guanine) → A (adenine). As a result of single frame-shift mutation, 521th amino acid was changed from G (glycine) → E (glutamic acid). So, we regarded the *ASP-LSL* gene as a candidate gene for the given phenotype of *asp-lsl*. To verify the mutation, 20 single plants were selected from the WT and *asp-lsl* from the F_2_ segregated population and verified using Tetra-Primer ARMS PCR.

### Knock out of *ASP-LSL* plants showed *asp-lsl* phenotype

**Fig. 7:**
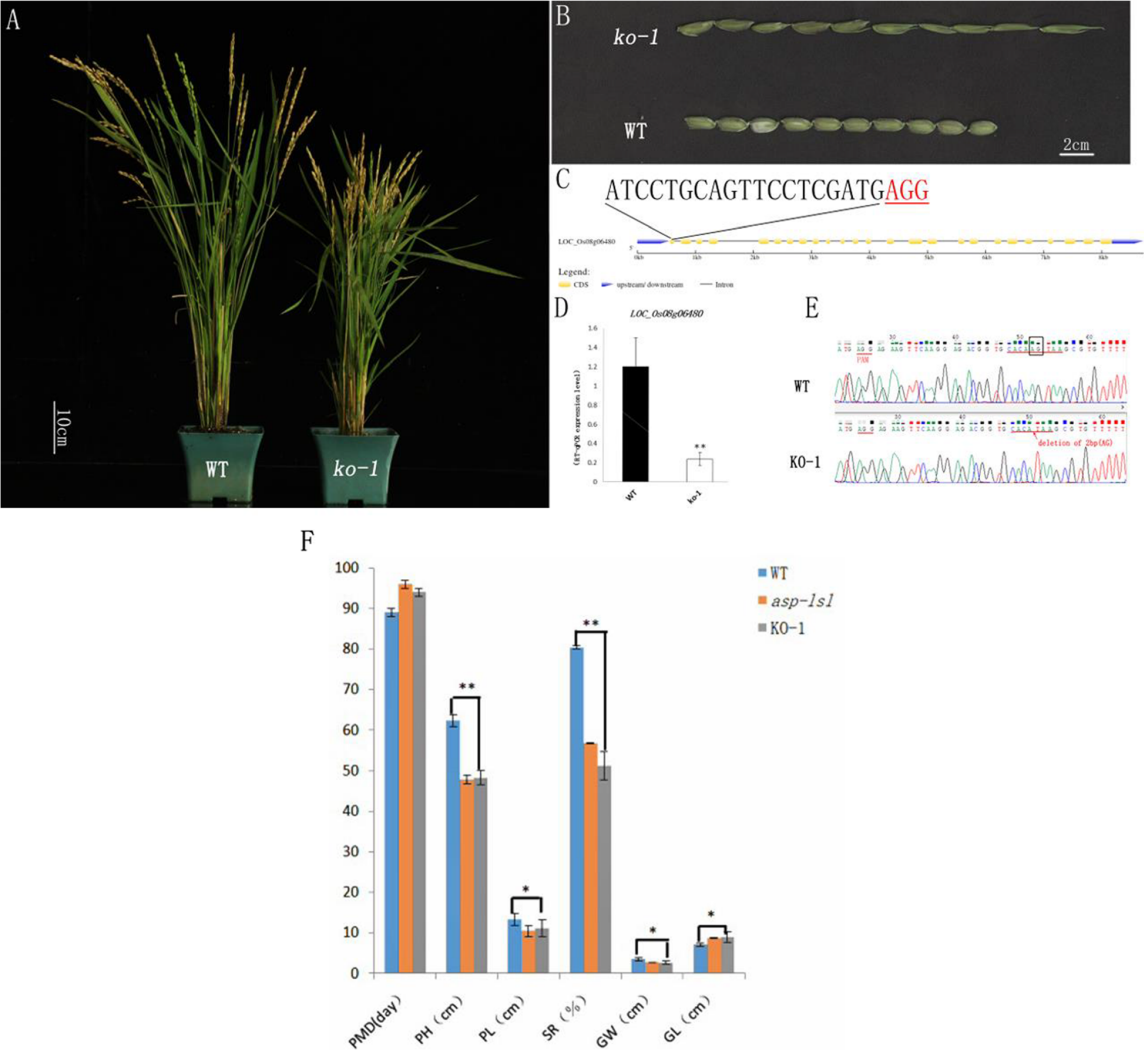
Phenotype of WT and knockout plant. (**A**) The phenotype of WT and *ko-1* (**B**) The phenotype of WT and *ko-1* floret. (**C**) The Targeted sequence of the first exon of *ASP-LSL* gene. (**D**) RT-qPCR analysis of *ASP-LSL* gene in WT and *ko-1* (**E**) Chromatogram (sequencing peaks) of WT and *ko-1***(F)** Agronomic traits comparison of WT, KO, and *asp-lsl.* Where PMD: Plant maturity days, PH: Plant height, PL: Panicle length SR: seed setting rate, GW: Grain weight GL: Grain length

In order to verify, the *ASP-LSL* gene is responsible for the above-mentioned mutant phenotype (Fig. 7A). A CRISPR/Cas9 vector targeted at the first exon (ATCCTGCAGTTCCTCGATGAGG) of *ASP-LSL*. The knock-out line-1 (*ko-1*) showed an increased length of sterile lemma, short plant height coupled with phenotype of *asp-lsl* (Fig. 7B). Meanwhile, we analyzed gene expression by RT-qPCR, and results showed a significant decrease in the expression *ASP-LSL* in knock-out lines (Fig. 7D). The chromatogram of *ko-1* showed a 2 bp deletion as compared to its WT (Fig. 7E). Number of days of maturity, plant height, panicle length, seed setting rate, grain width and grain length have been significantly decreased in *ko-1* as compared to WT (Fig. 7F).

### Phylogenetic analysis of *ASP-LSL*

**Fig. 8:**
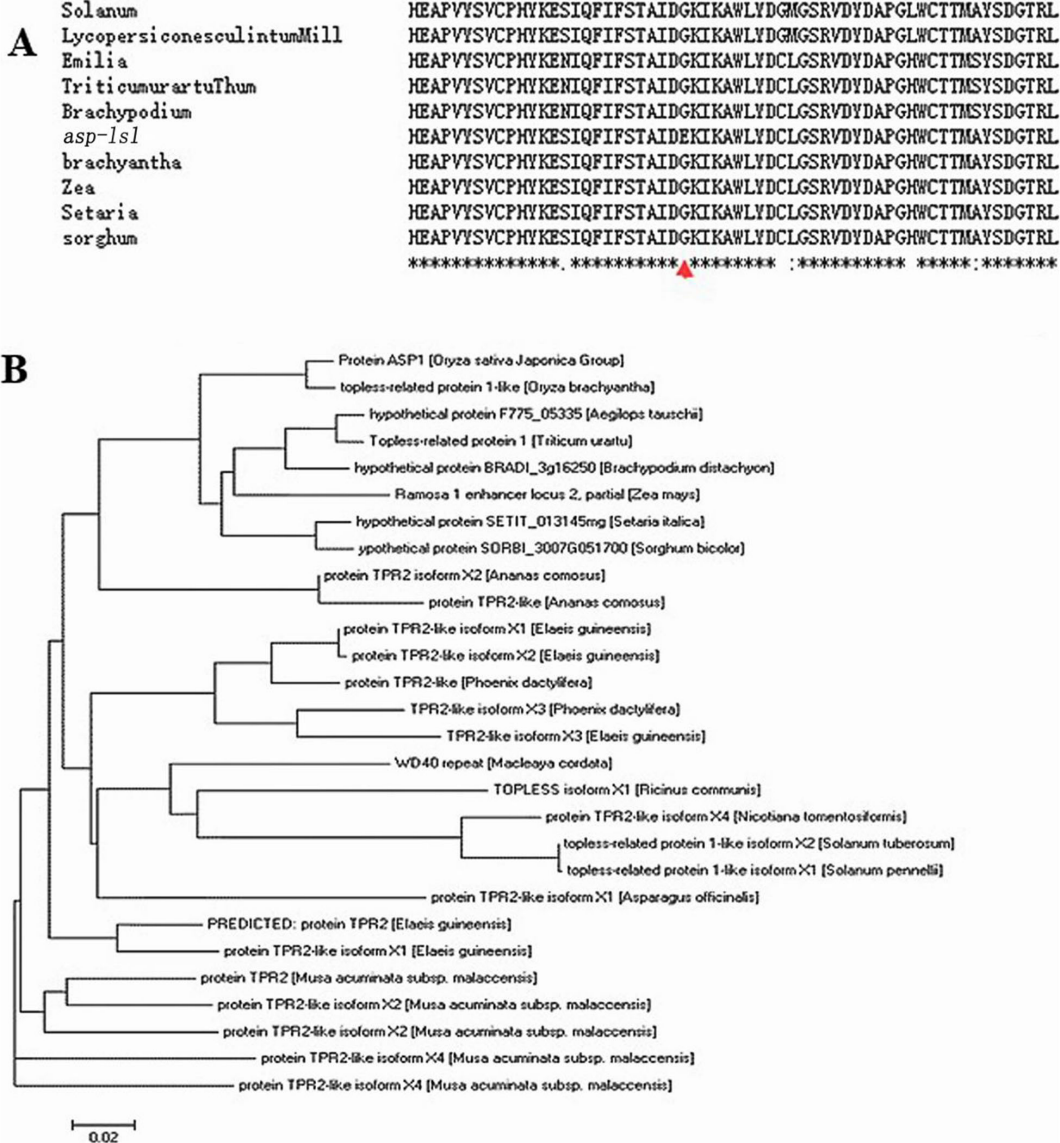
Alignment and phylogenetic tree of homologus proteins of *ASP-LSL* (**A**) Alignment analysis of the amino acid sequence of *ASP-LSL* (**B**) Phylogentic tree of homologus proteins of ASP-LSL

A complete comparison of amino acid sequence of *ASP-LSL* was used for construction of a phylogenetic tree at threshold of 70-99 %. As shown in the figure, all the reported mutations were occurred in a highly conserved action site, similar as in the mutant *asp-lsl* (Fig. 8A). The ASP-LSL protein has highest homology with a TOPLESS- protein in higher plants (Fig. 8B).

### Subcellular localization of *ASP-LSL*

**Fig. 9:**
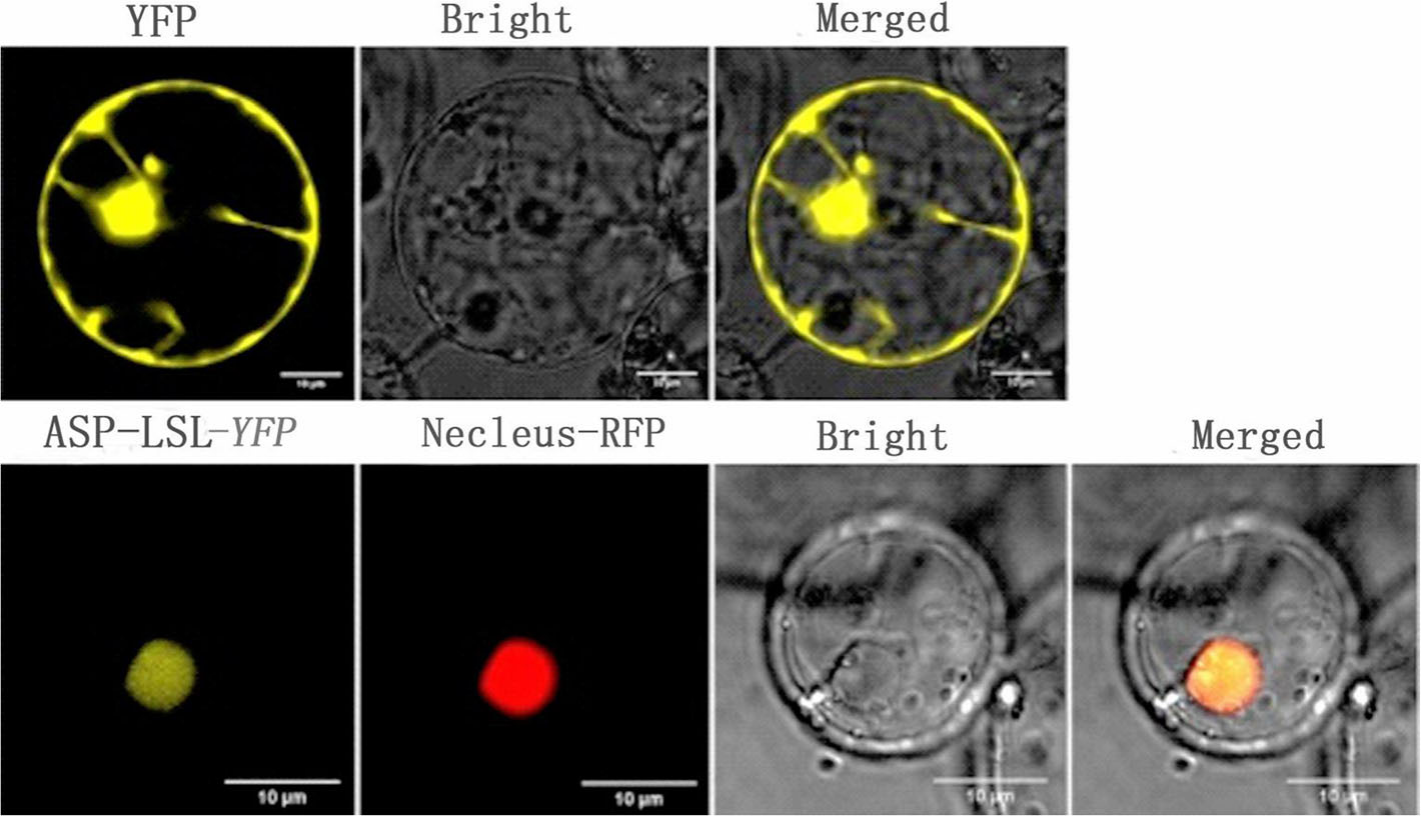
Subcellular localization of *ASP-LSL*

We constructed *ASP-LSL-YFP* and empty YFP vectors and transformed them into rice protoplasts. Compared with WT, nuclear colocalization of *ASP-LSL* revealed yellow fluorescence that overlaps with the red and formed an orange fluorescence as a nuclear localization signal (Fig. 9). Results of subcellular localization showed ASP-LSL is a nuclear protein.

### Relative expression of floral development-related genes in *ASP-LSL*

**Fig. 10:**
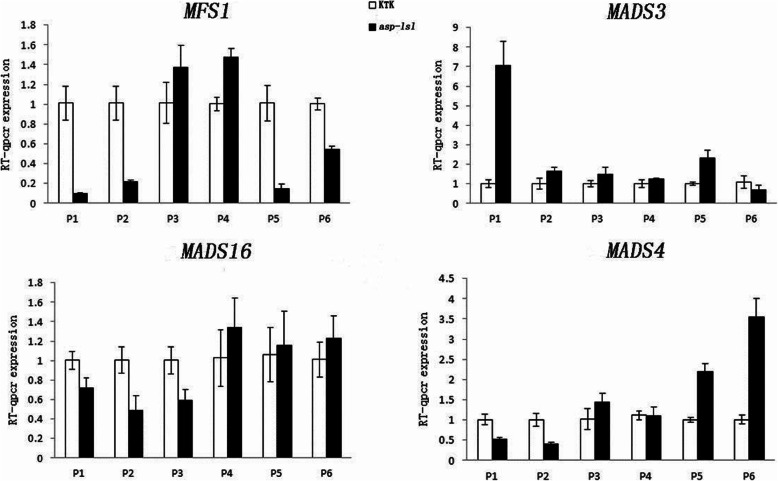
Relative expression analysis of different floral development-related genes in WT and *asp-lsl*

Development of *asp-lsl* was different than that of WT, to know which related genes were involved in expressing the different phenotype of *asp-lsl.* Young panicle at different growth periods were selected to detect the expression of different panicle development related genes. Different MADS genes e.g., *MFS1, OsMADS3, OsMADS4* and *OsMADS16* showed a significant change in *asp-lsl* as compared to WT. Quantitative expression of floral development-related genes showed different expression levels at different stages. The expression level of *MFS1* in the mutants was higher than that of WT at P3 and P4 stages (Fig. 10). *OsMADS4* and *OsMADS16* had higher expression levels in *asp-lsl* at the later stage of young panicle development, while *OsMADS3* showed the highest expression level at P1-P5. The sequence of primers used for relative expression analysis is given in Table 4.

**Table 4:** The primers used for the quantitative relative expression analysis of genes related to floret development

Primer	Sequence 5’ →3’
*OsMADS1*-F	TCTTGGTGAGGATTTGGGC
*OsMADS1*-R	CCTTGCTCTTCAGATCAAACAG
*OsMADS3-*F	AACGCAAACAGTAGGACCATAGTG
*OsMADS3*-R	CCCCTCTCATTCTCAACAACC
*OsMADS4-*F	AGCACAAGATGTTGGCTTTTAGGG
*OsMADS13-*F	ATGGGGAGGGGCAGGATTGAG
*OsMADS13-*R	TGCGCCTTCTTGTACCTGTCA
*OsMADS16-*F	CGAGGCGTACGAGACTCTGC
*OsMADS16-*R	ACCACGCGGAAGGCGAACAT
*DL-*F	CCCATCTGCTTACAACCGCTT
*DL-*R	GTTGGAGGTGGAAACCGTCG
*MFS1-*F	CGGCTCGTGATCTCGACACGTAC
*MFS1-*R	CACAGCCGGACCAGTGCTCTC

## Discussion

### *ASP-LSL* encodes a single recessive nuclear transactional repressor gene

In this study, an aberrant spikelet mutant was obtained by EMS mutagenesis of a wild cultivar, Kitaake. Through the inspection and comparison of agronomic traits, *asp-lsl* showed multiple defects including, the structure of spikelet and major agronomic yield traits e.g., plant height, number of tillers, seed setting rate, 1000-kernel weight, and number of effective grains per panicle were significantly compromised. Segregation and gene mapping analysis revealed its phenotypes were controlled by a single recessive nuclear. *LOC_Os08g06480* encodes a TPR/TPL nuclear transcriptional co-repressor. Transcriptional co-repressors have been already reported to play their role in controlling the spikelet, seed size and weight in rice and Arabidopsis [33, 50]. Phylogenetic analysis revealed that mutation was occurred in highly conserved site and have been reported in maize, sorghum and rice [1, 49]. This site can be targeted by molecular breeder to solve the specific problem of germination in hybrid rice [51]. Protein of this site may have specific binding activity to some other genes that regulate different traits including the development of lemma [48]. In recent years, many genes controlling flower development in rice have been cloned. Most of them are homologous to Arabidopsis and have a certain degree of conservation. These findings provide a basis for studying more about the regulation mechanism of rice flower development, without disturbing other major agronomic yield traits [51, 52]. In grasses, these homologous proteins can interact with ETHYLENE RESPONSE FACTOR (ERF), Jasmonic acid (JA), and IAA [47, 49]. Endogenous measurement of phytohormone contents suggested that *ASP-LSL* is sensitive to IAA that has an extensive role in plant growth and development activities. *ASP-LSL* also showed the action of IAA in regard to transition meristem fate and phyllotaxy during the vegetative phase [49, 53, 54]. The different phenotype of *ASP-LSL* could be a result of its molecular interaction with other spikelet development and auxin signaling pathway genes.

### *ASP-LSL* controls the novel phenotype in japonica cv. Kitaake

According to National Rice Data Center (ricedata.cn), *ASP-LSL* locus has already been reported in different articles. All the previous materials that were identified and reported so far belong to Nipponbare and Dongjin. In *asp-lsl*, site of mutation (521th amino acid Gly to Glu) is also novel, and this mutation has not been reported before. Moreover, all previous studies used T-DNA mutations, while we used EMS as a source of mutagenesis. Moreover, *asp-lsl* showed different salient features as compared to other mutants, which are given below.

1. *OsREL2* produced pleiotropic phenotype e.g., a greater number of secondary branches than primary branches and reduced number of floral organs etc., as compared to WT. T-DNA insertion mutant, *osrel2* also showed phenotype defects in panicle development. Contrary to *OsREL2, ASP-LSL* has an extra-long sterile lemma that bypasses the whole length of its spikelet. While in *osrel2* sterile lemma merely passes the half of the length of spikelet [47].
2. *OsLIS-L1* was a single recessive gene, reported to regulate pleiotropic phenotype and considered essential for fertility and internode elongation. *OsLIS-L1* encodes a protein that contains a WD40 motif, which is necessary for human brain development. *OsLIS-L1* was highly expressed in rice stems and panicles, but its expression in panicles showed dynamic changes at different developmental stages. Two independent alleles, *oslis-1-1* and *oslis-1-2* both showed reduced male gamete fertility, semi-dwarfism and shorter length of panicle. *OsLIS-L1* plays an important role in the elongation of the inverted internodes and the formation of male gametophytes [48]. The apparent difference of *OsLIS-L1* and *ASP-LSL* is the presence of a long sterile lemma in a later one.
3. *ASP1* encodes a transcriptional co-repressor, homologous to *TPL* of Arabidopsis and *REL2* in maize. Multiple phenotypic defects in *asp1* mutants were occurring, possibly due to the activation of multiple genes involved in meristem function. *Aps1* showed disorganized phyllotaxy and transition from branch to meristem formation was also compromised. The mutant with the loss of function of *ASP1* also revealed the bleaching of branches and spikelets. [55] A salient and novel feature of *ASP-LSL* with respect to *ASP1* is also the presence of long sterile lemma. In *asp1* the sterile lemma length reaches up merely to half of spikelet.

All of above three genes were located at the same gene locus as that of *ASP-LSL*, but mutant used in the current study contain conserved structure of the mutation site, a different source of germplasm, and the mode of mutagenesis was also different. Current study not only analyzes the biological function of this gene in more detail but also provide new germplasm resources for mutant bank pool. Current research was focused on the functional and phenotypic analysis of *ASP-LSL*. In the future, more in-depth work on the interaction and molecular regulation of *ASP-LSL* would be carried out.

## Conclusions

Current study reports the novel phenotype of *asp-lsl* that showed a significant decrease in plant height, number of tillers, thousand-grains weight, seed setting rate, spikelet length, kernel length and effective number of grains per panicle as compared to its WT. *Asp-lsl* showed delayed germination, deceased seed viability and shorter internodal distance than that of WT. Segregation and gene mapping analysis revealed its phenotypes were controlled by a single recessive nuclear. Subcellular localization of *ASP-LSL* revealed that its protein is localized in the nucleus. The knockout line of *ASP-LSL* produced a similar phenotype as that of *asp-lsl*. In the Hybrid seed production, spike germination is a prominent factor that affects the overall yield and milling quality of rice. Molecular breeders can manipulate the genes related to the pathway of long sterile lemma to solve the problem of spike germination with minimal damage to the other major agronomic traits.

## Methods

### Materials

A wild type (WT) japonica variety (Kitaake) was mutagenized by ethyl-methane sulfonate (EMS), and a mutant showing long sterile lemma coupled with other phenotypic defects was selected from M_2_ population and named as *asp-lsl*. Its phenotypic traits were found to be inherited stably after multiple generations of selfing. Two indica varieties Yixiang 1B and Kitaake were used as male parents and crossed with *asp-lsl* to construct F_2_ segregating populations. The materials used in experiment were planted under field conditions alternately at the experimental sites of Rice Research Institute, Sichuan Agricultural University in Lingshui (N18.47°, E110.04°), Hainan and Chongzhou (N30.67°, E103.67°), Sichuan, China. The sampling of material and experimental data was recorded with the permission and regular legislation of Rice Research Institute, Sichuan Agricultural University, China.

### Measurement of agronomic traits

At the maturity period of mutant and WT, 10 plants were randomly selected and data of plant height, ear length, grain length, grain width, thousand-grain weight, seed setting rate, inner and outer glume length and number of first branches, etc. were measured. The data were finally represented as an average of 10 individual plants for each of the corresponding trait index.

### Histology observations and identification of pollen fertility

Under normal field growth conditions, several primary differentiation stages of panicles were used for histology observation. The different tissue parts of mutant and WT panicles, lemma and glume at panicle elongation, grain filling and fully mature stage were fixed in FAA fixative at 4℃. After rinsing with series of 50 %, 70 %, and 80 % ethanol, and pictures were taken using a scanning electron microscope (FEI QUANTA 450). Fresh spikelets of mutant and WT with their pollens were observed under a microscope using potassium iodide staining.

### Dynamic observation of growth and development

To explore the difference between mutant and WT, a seed germination test was carried out in the laboratory. The specific parameters that were followed are given below.

#### Indoor germination test

One hundred healthy seeds of WT and mutant with their three biological repetitions were germinated at 37℃ in a light incubator. Germination rate was observed after 3 and 7 days, respectively.

#### Seed vigor examination

In order to verify the accuracy of the germination test results, 100 seeds were selected and cut down longitudinally and finally soaked in a 0.1 % tri-phenyltetrazolium chloride (TTC) solution for 3 h at 35 °C. After dyeing, TTC solution was poured out, and seeds were washed with clean water twice, and the stained number of embryos were counted.

#### Observation of seedling emergence time in an incubator

Fifty white seeds (stained) of WT and mutant were selected and sown in plastic pots using the soil culture method. Pots were kept under continuous observations, and emergence time from one-leaf to the five-leaves stage was recorded.

### Observation of root growth parameters

The WT and mutant plants were grown in a nutrient solution for 7 and 14 days were selected for root parameters observation. At the same time, the change of hypocotyl length was also measured in the absence of light at 32℃ in order to determine the relationship between *asp-lsl* and IAA. To explore its relationship with hormone transport and signal response, IAA solution was sprayed on the mutant plants grown for three days on culture. Whereas only distilled water was used as control treatment.

### Construction of the genetic map

For hundred fifty-six pairs of SSR markers constructed that were evenly distributed on all chromosomes of rice, were selected as primers, and the DNA of individual F_2_ populations was used as a template for PCR amplification. Bulk segregant analysis (BSA) was used to construct WT and mutant mixed DNA pools to screen out polymorphic differences through linkage markers. For confirmation of mutant phenotype, DNA of F_2_ progeny of single plant was used as DNA template. During primary gene mapping, the sequence of polymorphic loci was acquired from the biological database RGP (http://rgp.dna.affrc.go.jp/) and NCBI (https://blast.ncbi.nlm.nih.gov/Blast.cgi) and compared it with the sequence of model indica (9311) and japonica (nipponbare) varieties. SSR markers were designed using public databases e.g., RIS (http://rice.genomics.org.cn/rice/index2.jsp) and RGP (http://rgp.dna.affrc.go.jp/). To further fine map, indels markers were constructed and applied to the mutant DNA template. Chengdu Kinco Biological Co., Ltd synthesized the primers.

### PCR amplification

The specific quantity of ingredients used in PCR amplification was as follows; DNA (2 µl), F-Primer (1 µl), R-Primer (1 µl), dNTP (10mM) mixture (0.3 µl), Taq(5U/µl)0.2 µl, 10×Buffer mg^2+^ 2 µl and ddH_2_O (13.5 µl). The specific PCR amplification procedure was as follows: 95 °C pre-denaturation for 5 min; 95 °C denaturation for 30 s, 56 °C annealing for 30 s, 72 °C extension for 1 min, 34 cycles; 72 °C extension for 7 min, 4 °C storage for 1 min. The PCR products were separated on 3 % agarose gel electrophoresis and photographed under a Gel Documentation System (Bio-Rad Gel Doc 2000).

### Sequence analysis

Primer premier 6.0 and lingo 7.0 software were used primer designing and sequence analysis. As the target gene *ASP-LSL* has a relatively bigger size, we used the segmented amplification method and divided the gene into four fragments for easier amplification. PCR product was processed with Tiangen DNA recovery kit that is used for purification and separation. Recovered products were sent to Sichuan Qingke Biological Company (Ltd.) for gene sequencing.

### Genetic segregation analysis and knock out line development

The mutation sites were analyzed by dCAPS molecular marker primers, which were designed on (http://helix.wustl.edu/dcaps/dcaps.html). Twenty plants from F_2_ segregated population of WT and mutant were taken for DNA extraction, and PCR amplification was performed with a restriction endonuclease. A CRISPR/Cas9 vector targeted at the first exon of *ASP-LSL* was developed using BG Biotech Kit (BGK03), purchased from Hongzhou Bioge Biotechnology Co., Ltd and followed the protocol given by the manufacturer (http://www.biogle.cn/index/excrispr). *ASP-LSL* 19-bp PAM sequence was selected, and the Oligo sequence was generated online using BioGe website (www.bioge.cn). Target sites were selected to prevent off-target effects in the BLAST database (http://www.gramene.org/) for specificity. The synthesized oligo was dissolved in 10 ul of water. The following (buffer aneal 18ul, forward Oligo 1 ul, and reverse Oligo 1 ul) ingredients were mixed on ice and heated in a PCR apparatus at 95 °C for 3 min. The temperature was decreased to 20 °C at the rate of 0.2 °C/s. According to the manufacturer’s instruction, oligo dimer was constructed into CRISPR/Cas9 vector, each component (Oligo primer 1 ul, the CRISPR/Cas Vector 2 ul, enzyme mix 1 ul, 6 ul H_2_O) was evenly mixed on the ice and reacted in the PCR apparatus (20℃) for 60 min after mixing.

The recombinant system was transformed into *E. coli* according to the reference procedure. 10 ul of the reaction solution was added to 100 ul of competent cells, and then the mixture was mixed and placed in an ice bath for 30 min (do not shake during this period and keep standing strictly). The reaction mixture was gently taken out at 42℃ for 90 s, immediately placed on ice for 2 min, added 500 ul preheated LB liquid medium, and cultured at 37℃/200 RPM for 1 h. An appropriate amount of bacterial liquid was coated on the LB plate containing Kanamycin and placed upside down at 37℃ for overnight culture. The constructed vector was sent to Wuhan Boyuan Biotechnology Co., Ltd for genetic transformation.

### Quantitative relative expression analysis

The total RNA of mutant and WT roots, stems, leaves, and spikelets at the young panicle and early flowering stage were extracted using the RNA extraction kit. The purified RNA was reversed transcribed into cDNA. The sequence of a specific gene and its other homologous genes were quantified using primers. The template and reference gene (actin) was used in three biological repeats with two NTC (no template control) repeats. Quantitative expression was analyzed by Bio-Rad CFX Manager V2 that is based on ^△△^CT method.

### Bioinformatics analysis

Amino acid sequence homology was compared using NCBI (https://blast.ncbi.nlm.nih.gov/Blast.cgi) tool. Clustal X and MEGA software were used to perform comparison of complete sequence and construction of NJ evolutionary tree.

### Subcellular localization

The subcellular localization was predicted online using ChloroP 1.1 and TargetP 1.1 server. The full-length of *ASP-LSL* cDNA was amplified from its WT using specific primers (F: 5′ATGTCGTCGCTTAGCAGG-3′; R: 5′GACTTCTGGTTTGTTAGCT–3′) with BamHI at 5′-end and a SalI at 3′-end. The target fragments were cloned into pC1300-35 S-eYFP vector at the N-terminus of YELLOW FLUORESCENT PROTEIN (YFP). The constructs were transformed into rice protoplasts and incubated before the examination. YFP fluorescence signals were detected using a Laser Scanning Confocal Microscope (Nikon A1).

## Data Availability

The datasets supporting the conclusions of this article are included within the article.

## Abbreviations

A: Amyloplast
AP1: ACITVATOR PROTEIN 1
ASP1: ABERRANT SPIKELET AND PANICLE 1
asp-lsl: Aberrant spikelet-long sterile lemma
BG: Before grouting
CC: Companion cells
CRC: CRABSCLAW
CTK: Cytokinin
DL: DROOPING LEAF
EC: Epidermis cell
EMS: Ethyl-methane sulphonate
FBP: F-BOX PROTEIN
FM: Floral meristem
GA: Gibberellins
GL: Grain length
GW: Grain weight
IAA: Indole acetic acid
LHS1: LEAFY HULL STERILE 1
LOFSEP: LOSS OF TRANSCRIPTION FACTOR OF SEP
MFO1: MOSAIC FLRALORGANS 1
MFS2: MULTI-FLORET SPIKELET2
MS: Mature stage
OsLIS-L1: LISSENCEPHALY TYPE-1-LIKE-1
OsREL2: ROMOSA ENHANCER LOCI2
OsREL2: ROMOSA ENHANCER LOCI2
PAP2: PANlCLE PHYTOMER2
PH: Plant height
PI: PSEUDOGENE
PL: Panicle length
PMD: Plant maturity days
S: Stem
SC: Silicide cell
SEP: SEPALLATA
SM: Spikelet meristem
SPW1: SUPERWOMAN1
SR: Seed setting rate
STK: SERINE/THREONINE KINAS
TPL: TOPLESS
VBS: Vascular bundle sheath
Ve: Vessel
WT: Wild type
YP: Young panicle
